# X-linked Agammaglobulinemia Presenting with Multiviral Pneumonia

**DOI:** 10.7759/cureus.7884

**Published:** 2020-04-29

**Authors:** Yadis M Arroyo-Martinez, Michael Saindon, Jilmil S Raina

**Affiliations:** 1 Internal Medicine, University of South Florida, Tampa, USA; 2 Internal Medicine, Smolensk State Medical University, Smolensk, RUS

**Keywords:** hypogammaglobulinemia, xla, multiviral pneumonia

## Abstract

X-linked agammaglobulinemia (XLA) is a primary humoral immunodeficiency characterized by severe hypogammaglobulinemia and increased risk of infection. The genetic condition results from a mutation in the Bruton tyrosine kinase (BTK) gene located on the X chromosome leading to a near absence of B cells. Patients affected by XLA are most commonly predisposed to frequent and severe bacterial infections. However, here we report the case of a 20-year-old male with XLA who presented with viral pneumonia with multiple pathogens. This coexistence has been rarely reported. The patient received intravenous immunoglobulin therapy with noted significant improvement in the two weeks of follow-up. His clinical history supports the hypothesis of increased susceptibility to viral pathogens in the absence of immunoglobulin therapy. The humoral defect is the cornerstone of this phenomenon. This case presents the importance of multiviral causes for patients with recurrent episodes of pneumonia in an immunocompromised state.

## Introduction

X-linked agammaglobulinemia (XLA) is a primary humoral immunodeficiency which is characterized by severe hypogammaglobulinemia. This hypogammaglobulinemia results from a defect in the gene encoding* *Bruton tyrosine kinase (BTK) and leads to a near absence of CD19+ B cells and subsequent antibody deficiency [1,2]. Patients with XLA classically present with recurrent sinopulmonary infections and otitis media often secondary to encapsulated bacteria [2]. However, the coexistence of multiviral pneumonia in a patient with XLA has been rarely reported. We herein report the case of a young male with XLA who presented with multiviral pneumonia.

## Case presentation

**Figure 1:**
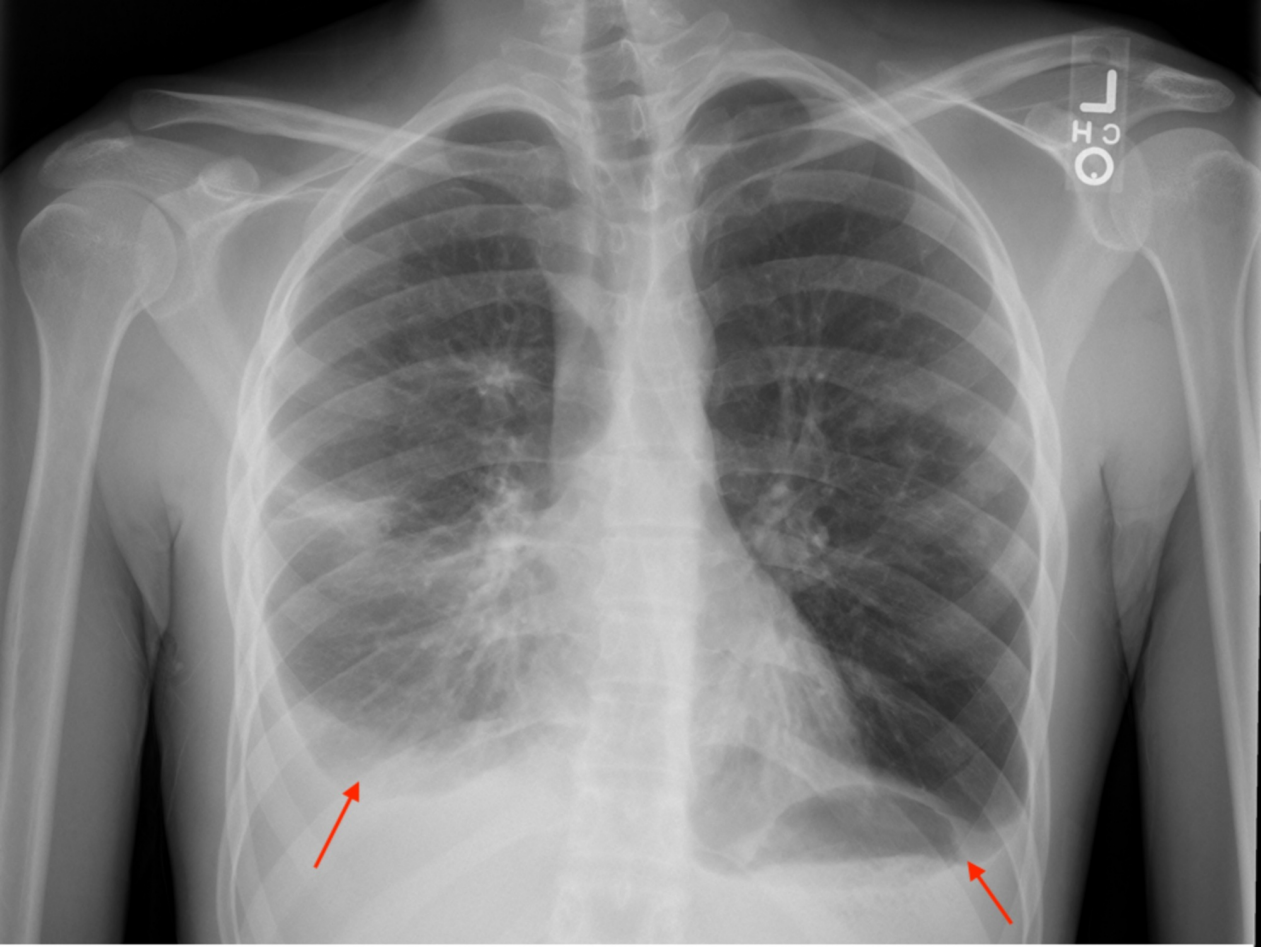
Chest x-ray with small to moderate size right pleural effusion (red arrow) and questionable opacities in the right middle lobe and anterior segment of the right upper lobe. Also note small left pleural effusion (red arrow).

We present a case of a 20-year old male with a past medical history of XLA, who presented in October with shortness of breath, cough, and pleuritic chest pain for approximately one week. Of note, the patient had been on intravenous immunoglobulins (IVIG) since diagnosis but had not received IVIG in the 12 months preceding this hospitalization due to loss to follow-up. He had a prior history of infection significant for multiple recurrences of bacterial pneumonia (last infection with *Moraxella catarrhalis* and *Streptococcus pneumoniae* one year prior to admission) and bronchiectasis. The patient had been briefly hospitalized overnight for cough and shortness of breath three to four weeks prior to this encounter. He reported receiving antibiotic therapy during that brief hospitalization but could not recall the specifics of his treatment regimen or final diagnosis. Initially on this admission, he was febrile to 39.5°C and tachycardic with a heart rate of 100-115 beats per minute. Labs were significant for a leukocytosis of 17.05 x 10^3^/µL with neutrophilic predominance and left shift. B- and T-cell subset analysis was significant for absolute CD4 cell count of 870/μL (500-2,600/μL), %CD4 of 63% (33%-66%), CD3 cell count of 1,340/μL (700-3,300/μL), and %CD19 (B cell) of 0% (4%-20%). Quantitative immunoglobulin levels were remarkable for IgG <108 (540-1,822 mg/dL), IgM <5 (22.0-240 mg/dL), and IgA <5 (63.0-484 mg/dL). A chest X-ray showed small to moderate right-sided and small left-sided pleural effusions and questionable opacities in the right lung (Figure 1).

**Figure 2:**
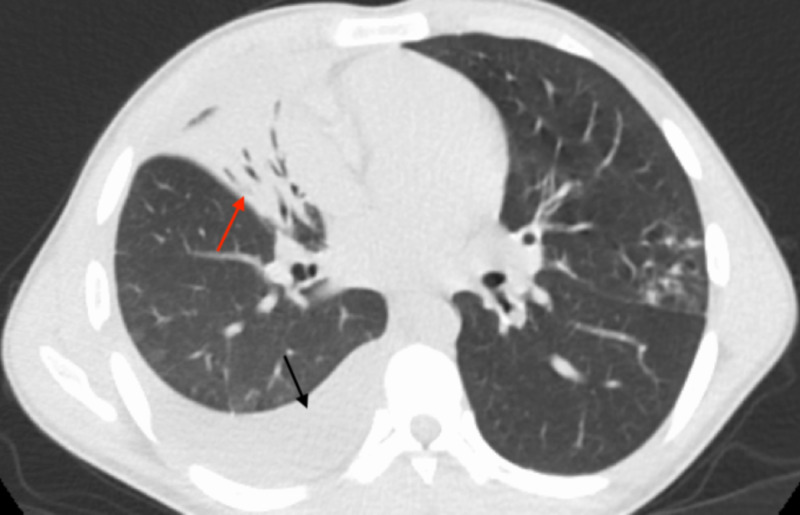
CT of the thorax demonstrating right middle lobe consolidation with air bronchograms and bronchiectasis (red arrow). Moderate-sized right pleural effusion (black arrow).

**Figure 3:**
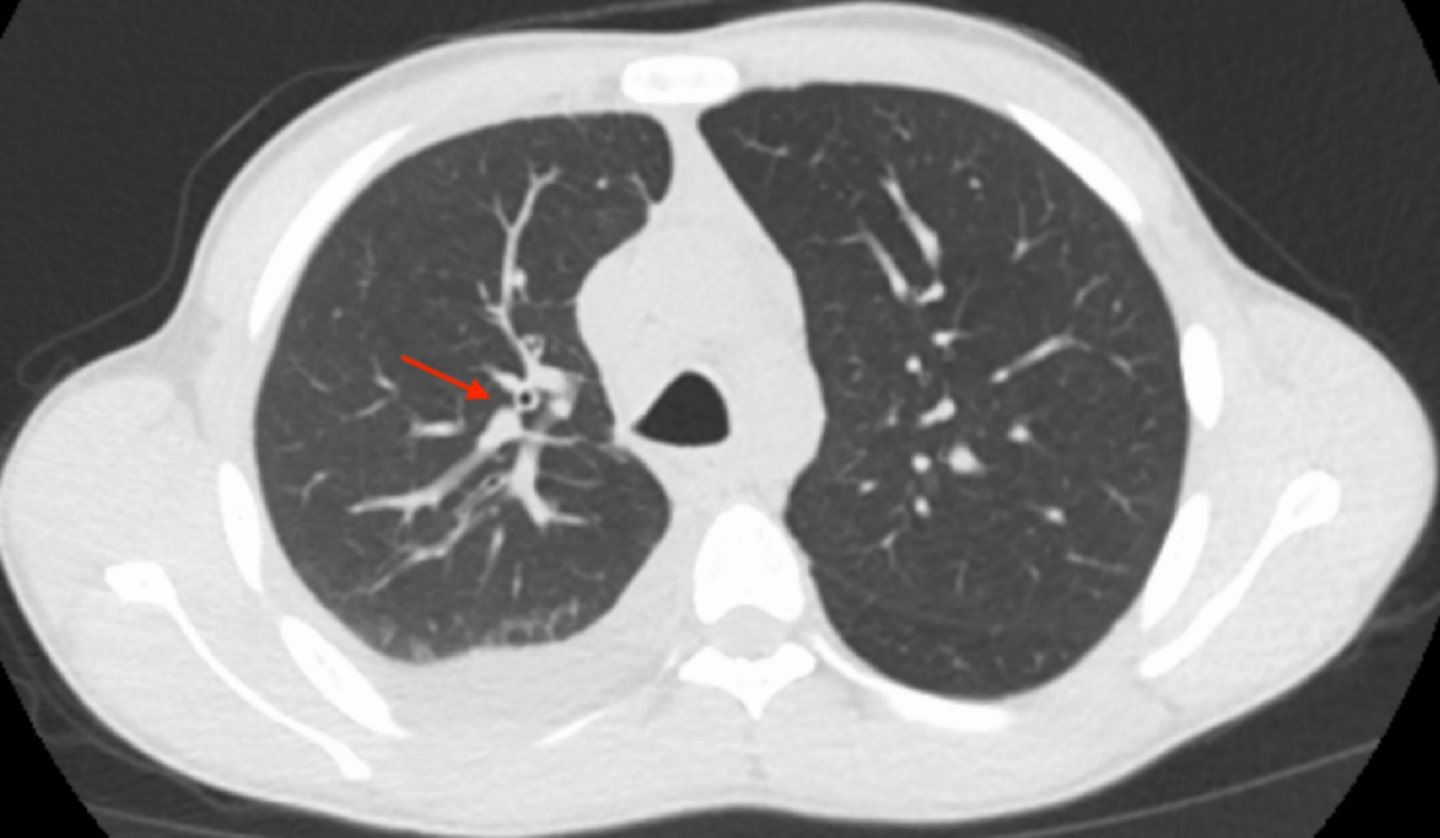
CT of the thorax demonstrating right middle lobe tree-in-bud nodularity and bronchiectasis (red arrow).

The patient was started on vancomycin and piperacillin/tazobactam and fluid resuscitated. A CT scan of the chest showed bronchopneumonia and moderate right-sided and trace left-sided pleural effusion (Figures 2, 3). Sputum culture showed mixed flora likely oropharyngeal, and blood cultures drawn prior to the initiation of antibiotic therapy were negative for pathogenic organisms. Subsequent bronchoscopy and right-sided thoracentesis were performed. Polymerase chain reaction (PCR) of the bronchial washings and pleural fluid were positive for cytomegalovirus (CMV), respiratory syncytial virus (RSV), and rhinovirus. CMV PCR bronchial washing had a viral load of 3,278, but CMV plasma PCR was negative. Bronchial washings were negative for *Mycobacterium tuberculosis*, *Mycobacterium avium-intracellulare*, *Nocardia*, *Legionella*, any fungal organisms, and any acid-fast bacilli. Pleural fluid analysis was consistent with an exudative effusion; however, all cultures were negative and no leukemia/lymphoma cell population was detected either. Serum antinuclear antibody, cyclic citrullinated peptide and rheumatoid factor were within the normal range. All antibiotics were discontinued following a seven-day course of therapy, and care was continued with supportive management. During this time, immunology was consulted, and the patient received IVIG with subsequent uptrend of his serum IgG level. The patient continued to improve clinically and was discharged home with appropriate follow-up. 

## Discussion

XLA is a hereditary immunodeficiency caused by mutations in the X chromosome in the gene encoding BTK and is characterized by recurrent severe infections [3]. The BTK gene encodes a cytoplasmic tyrosine kinase expressed in hematopoietic cell lineages [1]. The genetic mutation halts B-cell development at the pro-B cell-stage resulting in a congenital defect in B-cell production. This in turn leads to peripheral B-cell numbers of <1% and subsequent agammaglobulinemia [4,5]. 

These recurrent infections commonly occur after birth at around seven to nine months of age, when transplacental antibody titers begin to decrease and when the infant's body is unable to compensate for decreased levels of antibodies [3]. The diagnosis of XLA is based on low serum immunoglobulins, the absence of specific antibody response, lymphoid hypoplasia, and the near absence of B cells in the peripheral blood [3]. Given the rarity of the disease, an accurate estimate of the prevalence or incidence is difficult to obtain. However, results from a United States registry from 1988 to 1997 estimated an average of one in 379,000 total births per year or one in 190,000 male births per year of patients with XLA [2].

Patients with XLA are predisposed to frequent and severe respiratory infections mostly secondary to encapsulated bacteria. The most common pathogens are* Haemophilus influenzae*, *Streptococcus pneumoniae*, *Pseudomonas aeruginosa*, and *Moraxella catarrhalis* [6]. However, susceptibility to respiratory viruses has been rarely reported. Through online literature review only one study was encountered. The prospective study conducted by Kainulainen et al., in which two patients had XLA and 10 had common variable immunodeficiency, demonstrated the occurrence of respiratory tract viral infections in patients with primary hypogammaglobulinemia. The results showed that the 12 patients had a total of 65 episodes of acute respiratory tract infections in which the sputum of 54% of the episodes was positive for a viral respiratory tract infection. Rhinovirus was the most common virus. Upon the presentation of the respiratory tract symptoms, rhinovirus was found in the sputum in 32% of episodes either as a single virus 9%, together with bacteria 17%, or together with other viruses 6% [7].

The immunocompromised patient is at increased risk for lower respiratory tract infection due to community-acquired respiratory viruses when compared with the general population. RSV, influenza, parainfluenza, human metapneumovirus (hMPV), and adenovirus infections are of special importance. In immunocompromised host, the seasonal variability of each respiratory virus reflects that seen in the general population. As a result, RSV, influenza, and hMPV usually cause disease from November through April in the northern hemisphere; rhinovirus typically presents in the fall and spring; and adenovirus and parainfluenza occur mostly throughout the year [8]. It is also important to note the presence of Enteroviruses as they can cause persistent, often fatal infections in patients with hereditary or acquired defects in B lymphocyte function, such as patients with XLA [9].

The mechanisms of increased susceptibility to respiratory viral infections in hypogammaglobulinemic patients are not well understood. As mentioned previously, in patients with XLA the BTK gene is defective. This gene is known to contribute to Toll-like receptor (TLR) signaling, specifically TLR 8 and TLR 9. Both TLR 8 and TLR 9 are important in the activation of host defense against bacterial and viral infections [7]. Defective activation of TLRs leads to impaired production of proinflammatory cytokines, such as tumor necrosis factor alpha (TNF-a) and interleukin-6 (IL-6) [7]. The impaired production of IL-6 in patients with XLA may contribute to their increased susceptibility to respiratory viral infections.

Immunoglobulin replacement therapy is indicated for primary humoral immunodeficiencies that consist of absent or deficient antibody production [10]. Immunoglobulin therapy has increased the life expectancy and decreased the number of pulmonary infections in patients with XLA. Patients who are compliant in receiving IVIG therapy will generally benefit from fewer hospitalizations and lessened incidence of pulmonary insufficiency. Of note, prophylaxis against certain nonbacterial infections, including enteroviral infections, may require more aggressive dosing of immunoglobulin replacement therapy [11].

## Conclusions

This case provides an important example and assists in promoting awareness of patients with primary hypogammaglobulinemia presenting with a severe viral respiratory infection. In these patients keeping a viral etiology for symptoms in the differential diagnosis can prove beneficial as identifying the causative pathogen early in the clinical course will help to dictate appropriate management. Specifically, limiting exposure to unnecessary antimicrobial agents can help to reduce iatrogenic complications and help to avoid prolonged hospital stays. This case also serves to reinforce the importance of IVIG therapy to substantially reduce the number and severity of pulmonary infections in these patients.
